# Large numbers of explanatory variables: a probabilistic assessment

**DOI:** 10.1098/rspa.2017.0631

**Published:** 2018-07-04

**Authors:** H. S. Battey, D. R. Cox

**Affiliations:** 1 Department of Mathematics, Imperial College London, London SW7 2AZ, UK; 2 Nuffield College, University of Oxford, Oxford OX1 1NF, UK

**Keywords:** genomics, regression analysis, sparsity

## Abstract

Recently, Cox and Battey (2017 *Proc. Natl Acad. Sci. USA* **114**, 8592–8595 (doi:10.1073/pnas.1703764114)) outlined a procedure for regression analysis when there are a small number of study individuals and a large number of potential explanatory variables, but relatively few of the latter have a real effect. The present paper reports more formal statistical properties. The results are intended primarily to guide the choice of key tuning parameters.

## 1. Introduction

We consider studies of dependence in which there are relatively few individuals on each of which a large number of potential explanatory variables are measured. Genetic microarray experiments are a prime example. For progress, an implicit or explicit assumption of sparsity is required, namely that the great majority of the explanatory variables have no effect. We call explanatory variables with an effect on response signal variables, and those with no effect noise variables.

Powerful procedures for analysis emanate from the lasso [1] (for a discussion of the mathematical aspects, see [2]). They aim to uncover a single small set of signal variables effective for prediction. It is possible, however, that many different choices of explanatory variables are essentially equally effective and have quite different subject–matter implications. Cox & Battey [3] outline a different approach aiming to specify those small sets of potential signal variables that give essentially the same fit; choice between different sets requires either more data or specific subject–matter information. Their procedure also allowed informal checks standard in much statistical work, such as those for nonlinearity, interaction or anomalous individuals.

In the present paper, we outline a probabilistic base for such an analysis. The emphasis is on how the procedure would perform under various idealized scenarios, as such providing guidance on the choice of key tuning parameters. The focus is on two key aspects at each stage and in the overall process of analysis. What is the probability that a signal variable is falsely discarded? How many of the variables ultimately suggested as potentially important are in fact noise variables? Our objective is essentially to calibrate the analytical procedure by specifying its behaviour under idealized conditions, not to set up a procedure to achieve preassigned error rates.

The ideas involved apply rather generally, for example, to likelihood-based fitting of logistic models for binary data.

## 2. Broad outline of procedure

Suppose that *v* variables are to be assessed for their explanatory power for a response variable. It is assumed that *v* is roughly *k*^*d*^ for *k* ≤ 15 and *d* a small positive integer. In the method as outlined by Cox & Battey [3], the indices of these variables are arranged in a *k* × *k* × *k* cube for *d* = 3 or *k* × *k* × *k* × *k* hypercube for *d* = 4, etc. Sets of variables are then selected for test *k* at a time by traversing the cube by rows, columns, etc. Every variable thus has associated with it *d* sets of (*k* − 1) companion variables from each time the traversal of the cube passes through that variable. The explanatory power of each variable is assessed alongside its *d* sets of companions and, on the basis of those analyses, variables are either discarded or retained according to a suitable decision rule. For instance, a variable might be retained if it is among the two most significant in at least *d*/2 of the *d* analyses in which it appears.

This is repeated with successively lower dimensional hypercubes until ideally fewer than 20 variables remain on which informal diagnostic checks are then performed.

The final phase of the analysis is to find small subsets of variables among the augmented set of retained variables and any square or interaction terms suggested at the informal phase. See Cox & Battey [3] for a more detailed description of the exploratory and subset selection phases.

## 3. Specification

At the *i*th stage of the process, let there be respectively *v*_*Si*_ signal variables and *v*_*Ni*_ noise variables. In the specific example below, *i* = 0, 1, 2. Thus of the *v*_*S*0_ signal variables initially present, *v*_*S*2_ are chosen at the end for detailed study. Given *v*_*S*0_, the numbers of signal variables chosen at the first and second stage have binomial distributions with parameters *θ*_*S*1_ and *θ*_*S*2_ = *θ*_*S*1_*θ*_*S*2.1_, where, in particular, *θ*_*S*2.1_ is the conditional probability, given that a specific signal variable is chosen at the first stage, that it is chosen again at the second stage. A different specification is needed for the noise variable because, initially at least, there are a large number of them. In particular, the *v*_*Ni*_ have Poisson distributions with means *μ*_*Ni*_. We write *ϕ*_*N*2.1_ = *μ*_*N*2_/*μ*_*N*1_ for the probability that a noise variable retained after step 1 is still retained at step 2.

A summary of the properties of a two-stage procedure is thus provided by *θ*_*S*2_ and *μ*_*N*2_, the probability that a signal variable is retained and the expected number of noise variables not rejected.

We now study these in order to compare different strategies of analysis.

## 4. Some reduction strategies

We compare three possible approaches to each analysis of the first stage reduction. Thus, we may

— take the single variable with highest score (most significant);
— take the two variables with highest scores; and
— take all those variables, if any, whose scores exceed a threshold.

Significance tests based on the Wald, score or likelihood ratio statistics are natural choices. The former does, however, have the disadvantage of not being parameterization invariant.

In a set of *k* variables chosen at random for test, the number of signal variables has a Poisson distribution of mean *kv*_*S*0_/*v*, where *v* = *v*_*S*0_ + *v*_*N*0_ and *v*_*S*0_ is assumed modest, and hence has two or more signal variables with probability approximately *k*^2^*v*^2^_*S*0_/(2*v*^2^). This is small for the situations to be considered and does not affect the qualitative comparison of procedures. It is, therefore, ignored in later calculations.

It is convenient to phrase the initial discussion in terms of significance tests and their associated *p*-values. For noise variables, these are uniformly distributed on (0, 1), and for signal variables their density can be modelled as (1 − *γ*)*x*^−*γ*^, where 0 < *γ* < 1.

In the following calculations of the probability *𝜗*^(*j*)^ that the *j*th procedure listed above chooses the signal variable, it is convenient to use Stirling's formula in the form that for large *k* and fixed *a* the ratio *Γ*(*k* + *a*)/*Γ*(*k*) is close to *k*^*a*^.

If only the most significant out of *k* is chosen, the probability that the signal variable is taken is

4.1 $$\vartheta^{\left(1\right)}=\int_{0}^{1}dx(1-\gamma )x^{-\gamma }{\left(1-x\right)}^{k-1}=\frac{\Gamma (2-\gamma )}{k^{1-\gamma }},$$

after simplifying by Stirling's formula. Note that the Gamma function is close to 1 over the range of interest. If *γ* = 0, there is no distinction between signal and noise variables and the notional signal variable is selected with probability 1/*k*, that is, essentially at random.

If now we take the two most significant values out of *k*, then *𝜗*^(2)^ = *𝜗*^(1)^ + *𝜗*^(2.1)^, where

$$\vartheta^{\left(2.1\right)}=\int_{0}^{1}dx(k-1)x(1-\gamma )x^{-\gamma }{\left(1-x\right)}^{\left(k-2\right)}.$$

This is approximately

4.2 $$\frac{\left(1-\gamma )\Gamma (2-\gamma \right)}{k^{1-\gamma }}.$$

**Figure 1.**
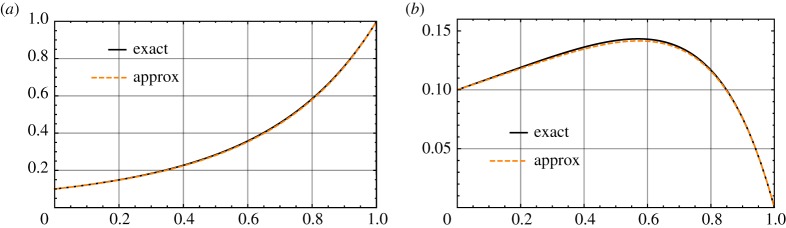
Exact and approximate values of *𝜗*^(1)^ (*a*) and *𝜗*^(2.1)^ (*b*) over *γ*∈ (0, 1) for *k* = 10. (Online version in colour.)

That is, approximately, *𝜗*^(2.1)^/*𝜗*^(1)^ = 1 − *γ*. The difference between *𝜗*^(2)^ and *𝜗*^(1)^ is maximized at approximately *γ*_0_ = (log*k* − 1)/log*k*, at which *𝜗*^(2.1)^ is *Γ*{(log*k*)^−1^}/(*e*log*k*) (figure 1).

If we were to set a critical level at *α*, then the corresponding probability for a signal is

$$\vartheta^{\left(3\right)}=\int_{0}^{\alpha }(1-\gamma )x^{-\gamma }\;dx=\alpha^{1-\gamma },$$

so that the equalities *𝜗*^(3)^ = *𝜗*^(1)^ and *𝜗*^(3)^ = *𝜗*^(2)^ are achieved for any *γ* by setting, respectively, *α* = *k*^−1^*Γ*(2 − *γ*)^1/(1−*γ*)^ ≈ *k*^−1^ and

$$\alpha =k^{-1}{\left\{\Gamma (2-\gamma )(2-\gamma )\right\}}^{1/(1-\gamma )}\approx k^{-1}{\left(2-\gamma \right)}^{1/(1-\gamma )}.$$

This shows that strategy 3 with *α* = 1/*k* and strategy 1 are equivalent as this choice also makes *μ*^(3)^ = *μ*^(1)^. By contrast, (2 − *γ*)^1/(1−*γ*)^ > 2 for all 0 < *γ* < 1, meaning that, for the same probability of selecting a signal variable, strategy 3 necessarily selects more noise variables than strategy 2 and, therefore, is strictly inferior.

In replacing the second strategy by the first, *μ*_*N*1_ and consequently *μ*_*N*2_ are reduced by roughly a factor of four independently of *γ*. However, the penalties of including noise variables are mainly incidental in view of the exhaustive search over models that must later take place. The associated computational burdens are reasonable unless *μ*_*N*2_ is, for example, 20 or more, and so strategy 2 would normally be preferred for its higher *θ*_*S*1_.

The probability *θ*^(*j*)^_*S*1_ that the *j*th procedure retains a signal variable through the first stage is *θ*^(*j*)^_*S*1_ = *𝜗*^(*j*)3^ + 3*𝜗*^(*j*)2^(1 − *𝜗*^(*j*)^) and so, approximately,

4.3 $$\theta_{S1}^{\left(1\right)}-\theta_{S1}^{\left(2\right)}=(1-\gamma )k^{2\gamma -3}[3(\gamma -3)k+\{7+(\gamma -5)\gamma \}k^{\gamma }]\leq 0,$$

with equality at *γ* = 1, is the reduction in survival probability of signal variables from replacing the second procedure by the first. Here we have approximated by treating analyses in which the same variable appears as independent. The difference (4.3) is approximately

4.4 $$\frac{1+3\log k\{1-e(1+2\log k)+\log k\}}{{\left(e\log k\right)}^{3}},$$

at the point of maximum distinction *γ* = *γ*_0_, and as the slowest decaying term in (4.4) is of the order of 1/(*e*^2^log*k*) this shows the potential advantages of strategy 2 over strategy 1 to decrease only very slowly with *k*.

A less general but more conventional formulation is to assume a normal theory linear model. Define Δ to be signal strength multiplied by the square root of the sample size. The values of *𝜗*^(^*j*) are then

$$\begin{array}{rl} \vartheta^{\left(1\right)} & =\int \phi (x-\Delta )\Phi^{k-1}(x)\;dx,\\ \vartheta^{\left(2\right)} & =\vartheta^{\left(1\right)}+\int (k-1)\phi (x-\Delta )\Phi^{k-2}(x)\Phi (-x)\;dx,\\ \vartheta^{\left(3\right)} & =\Phi (\Delta -\kappa^{*}), \end{array}$$

where *ϕ*(*x*) and *Φ*(*x*) are the standard normal probability density and cumulative distribution function at *x*, respectively. The threshold in the third strategy is, by convention, calibrated as the upper quantile κ* of the standard normal distribution. We thereby approximate *𝜗*^(1)^ and $\vartheta^{\left(2.1\right)}≜(k-1)\int \phi (x-\Delta )\Phi^{k-2}(x)\Phi (-x)\;dx$ and show that the two formulations are qualitatively equivalent.

The integral in *𝜗*^(2.1)^ is negligible over $R^{-}$ for Δ > 0 and over $R^{+}$ is of the form of a generalized Laplace integral for Δ fixed, that is,

4.5 $$\int_{0}^{\infty }g(x,\nu )\exp\{\nu h(x,\nu )\}\;dx,$$

where *g*(*x*, *ν*) is uniformly bounded in *x* as ν→∞ and *h* has a single maximum, *x*_0_(*ν*), which varies with *ν*. Specifically we take *g*(*x*) = *ϕ*(*x* − Δ), *ν* = *k* − 2 and *h*(*x*, *ν*) = log*Φ*(*x*) + log*Φ*( − *x*)/*ν*. The integral *𝜗*^(2.1)^ is thus approximable by the method of Laplace (e.g. [4, pp. 60–65]). The idea in outline is that, for large *ν*, by far the greatest contribution to the integral comes from a neighbourhood of *x*_0_. The integral is evaluated by expanding *g* and *h* in a neighbourhood of *x* = *x*_0_, leading to

4.6 $$\vartheta^{\left(2.1\right)}=(k-1)g(x_{0},\Delta )\exp\{h(x_{0},\nu )\nu \}{\left\{-\frac{2\pi }{h^{\prime \prime }(x_{0},\nu )\nu }\right\}}^{1/2}+O\left(\frac{1}{\nu^{2}}\right),$$

where the symbol^′^ denotes the partial derivative with respect to the first argument. It is customary when constructing Laplace integrals to define *h* such that its maximum is independent of *ν*. This is not possible here as, without the contribution of the *Φ*( − *x*) term, *h* has no sharp maximum. That the form in (4.5) is permissible is discussed by Copson [5, pp. 42–47].

The second derivative at *x* is

$$h^{\prime \prime }(x,\nu )=x\phi (x)\left\{\frac{\Phi (x)-\Phi (-x)\nu }{\Phi (-x)\Phi (x)\nu }\right\}-\phi^{2}(x)\left\{\frac{\Phi^{2}(-x)\nu +\Phi^{2}(x)}{{\left\{\Phi (-x)\Phi (x)\right\}}^{2}\nu }\right\}$$

and the unique maximizer *x*_0_ of *h* satisfies

4.7 $$0=\phi (x_{0})\left\{\frac{1}{\Phi (x_{0})}-\frac{1}{\Phi (-x_{0})\nu }\right\}.$$

Let ${\tilde{x}}_{0}≜-\Phi^{-1}(1/k)$. As $\Phi ({\tilde{x}}_{0})=(k-1)/k=1+O(k^{-1})$, we have $x_{0}≃{\tilde{x}}_{0}$, leading to

4.8 $$h^{\prime \prime }(x_{0},\nu )≃-\phi^{2}\left\{\Phi \left(\frac{1}{k}\right)\right\}k,\;(k\to \infty ),$$

and an asymptotic approximation to *𝜗*^(2.1)^ is

4.9 $$\begin{array}{rl} & \frac{\sqrt{\left(2\pi \right)}{\left(k-1\right)}^{k}}{k^{k+1}}\frac{\phi (x_{0}-\Delta )}{\phi \{\Phi^{-1}(1/k)\}}\\ & \;=2\pi \frac{{\left(k-1\right)}^{k}}{k^{k+1}}\phi (\Delta )\exp\left\{-\Delta \Phi^{-1}\left(\frac{1}{k}\right)\right\},\;(k\to \infty ). \end{array}$$

Equation (4.9) has a unique maximum at Δ_0_(*k*) = − *Φ*^−1^(1/*k*) of

4.10 $$\exp\left[\frac{1}{2}{\left\{\Phi^{-1}\left(\frac{1}{k}\right)\right\}}^{2}\right]\frac{{\left(k-1\right)}^{k}}{k^{k+1}}\sqrt{\left(2\pi \right)}.$$

**Figure 2.**
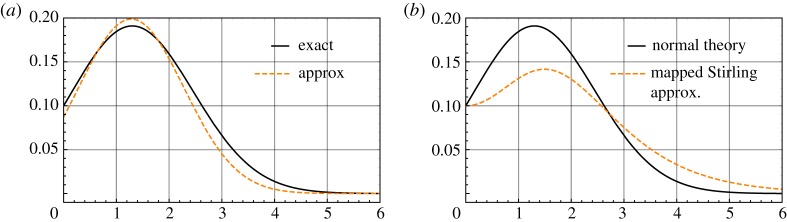
(*a*) Exact and approximate values of *𝜗*^(2.1)^ as functions of Δ for *k* = 10; (*b*) *𝜗*^(2.1)^ as a function of Δ for *k* = 10 in the normal theory formulation and in the *p*-value formulation with *γ* = {cosh(Δ) − 1}/cosh(Δ). (Online version in colour.)

Figure 2*a* shows the accuracy of the approximation (4.9) for *k* = 10.

The qualitative implications of the normal theory formulation are equivalent to those of the formulation in terms of *p*-values. For instance, at Δ = 0, which corresponds to *γ* = 0, equation (4.9) is $\sqrt{\left(2\pi \right)}{\left(k-1\right)}^{k}k^{-(k+1)}$. This is 1/*k* to two decimal places for *k* sufficiently large. If *γ* is redefined as *γ* = {cosh(Δ) − 1}/cosh(Δ), then equation (4.2) has a unique maximum at sech^−1^(1/log*k*), which, like Δ_0_(*k*), is a very slowly growing function of *k*. Figure 2*b* shows the approximation in equation (4.2) as a function of Δ with *γ* redefined as described. Any mapping Δ↦*g*(Δ) such that $g:R^{+}\to (0,1)$ is monotonically increasing with *g*(0) = 0 and *g*(*x*) ≈ 1 for *x* > 4 gives essentially the same qualitative conclusions.

## 5. Relative severity of each stage

We now consider the relative advantages of

— a mild first stage and severe second stage
— a severe first stage and a mild second stage,

where, for clarity of exposition, ‘mild’ and ‘severe’ are associated with the same quantitative values in both versions. In particular, let 1 > *𝜗*_H_ > *𝜗*_L_ > 0, where *𝜗*_H_ and *𝜗*_L_ are the probabilities that a signal variable is retained in a single analysis of a mild and severe reduction, respectively. Let *θ*^HL^_*S*2_ and *θ*^LH^_*S*2_ denote the overall survival probabilities of the first and the second of these procedures, respectively. To demonstrate that

$$\begin{array}{rl} \theta_{S2}^{\mathrm{HL}} & =\{\vartheta_{H}^{3}+3\vartheta_{H}^{2}(1-\vartheta_{H})\}\{\vartheta_{L}^{2}+2\vartheta_{L}(1-\vartheta_{L})\}\\ & >\{\vartheta_{L}^{3}+3\vartheta_{L}^{2}(1-\vartheta_{L})\}\{\vartheta_{H}^{2}+2\vartheta_{H}(1-\vartheta_{H})\}=\theta_{S2}^{\mathrm{LH}}, \end{array}$$

or equivalently

$$\frac{\vartheta_{H}(2-\vartheta_{H})}{\vartheta_{L}(2-\vartheta_{L})}<\frac{\vartheta_{H}\{\vartheta_{H}(3-2\vartheta_{H}\}}{\vartheta_{L}\{\vartheta_{L}(3-2\vartheta_{L}\}},$$

suppose for a contradiction that

5.1 $$\frac{\vartheta_{H}}{\vartheta_{L}}\leq \frac{\left(2-\vartheta_{H}\right)}{\left(2-\vartheta_{L}\right)}\frac{\left(3-2\vartheta_{L}\right)}{\left(3-2\vartheta_{H}\right)}=1+\frac{\vartheta_{H}-\vartheta_{L}}{6-\{3\vartheta_{L}+2\vartheta_{H}(2-\vartheta_{L})\}}.$$

The supremum of 3*𝜗*_L_ + 2*𝜗*_H_(2 − *𝜗*_L_) is 5, attained only in the limit as $\vartheta_{L}\to \vartheta_{H}\to 1$. But 5 < 6 − *𝜗*_L_ because *𝜗*_L_ < 1 and so equation (5.1) implies

$$\frac{\vartheta_{H}}{\vartheta_{L}}<1+\frac{\vartheta_{H}-\vartheta_{L}}{\vartheta_{L}}=\frac{\vartheta_{H}}{\vartheta_{L}},$$

a contradiction, and we conclude that *θ*^HL^_*S*2_ > *θ*^LH^_*S*2_. That is, the first procedure gives a higher probability of a signal variable being retained than the second. The same argument shows that the first procedure results also in a higher probability of noise variables being retained.

## 6. A simple example

The performance of various stages of the procedure may be investigated as follows. Generate *n* = 10^2^ replicates of *v* = 10^3^ variables from a normal distribution of zero mean and covariance matrix *PΣP*^−1^, where *P* is a permutation matrix and *Σ* is an identity matrix with one diagonal block replaced by a correlation matrix of dimension *v*_*S*0_ + *v*_*C*0_ and equal correlation *ρ*. For each replicate, *v*_*S*0_ of the *v*_*S*0_ + *v*_*C*0_ correlated variables is multiplied by a constant signal and added, together with standard normal noise. Conditional on a realization $x_{S}$ of the *v*_*S*0_ signal variables, the resulting response variable is then normally distributed of mean $\gamma^{T}x_{S}$ and unit variance, where *γ* is a *v*_*S*0_ vector of constants. Arrange the indices of the *v* variables in a 10 × 10 × 10 cube. The rows, the columns, etc. of the cube form 3*k*^2^ = 300 sets, each of *k* = 10 variables. Reduction is performed by taking the top two scoring variables in each analysis in the first stage and by taking all those exceeding the threshold of a 0.1% level test in the second stage. Finally, find small subsets of variables that give adequate fit using a likelihood ratio test against the comprehensive model.

Summaries estimated from 500 Monte Carlo replications are reported in table 1. The general conclusion is that such correlation slightly degrades the probability of a signal variable being retained and increases the number of false models not rejected. The comprehensive model in the likelihood ratio test is taken as the model with all variables retained through the reduction phase. Having been selected in the light of the data, it achieves a better fit to the data than an arbitrary model embedding the one to be tested and so the probability of the true model being accepted, conditional on it having been retained, is lower than the nominal coverage probability of the likelihood ratio test. A simple way to recover the conditional nominal coverage is to split the sample. Table 1 reports the improvement in coverage probability and the associated increase in the number of false models not rejected from using 70 observations for reduction and the remaining 30 to construct the final set of low-dimensional models.

**Table 1.** S is the true set of signal variables, $\hat{S}$ is the set of variables surviving the reduction phase, M is the set of low-dimensional models whose likelihood ratio test against the comprehensive model is not rejected at the 1% level. Empirical standard errors in parenthesis.

				$\mathrm{pr}(S\subseteq \hat{S})$			pr(S∈M)		E|M∖S|	
*v*_*S*0_	*v*_*C*0_	*ρ*	$\frac{\mathrm{signal}}{\mathrm{noise}}$	lasso	full sample	split sample	full sample	split sample	full sample	split sample
1	1	0.9	1	1.00 (0.04)	1.00 (0.00)	1.00 (0.00)	0.57 (0.50)	0.99 (0.08)	6.8 (9.0)	15.7 (30.0)
1	1	0.9	0.6	0.95 (0.21)	0.96 (0.21)	0.74 (0.44)	0.45 (0.50)	0.74 (0.44)	5.4 (6.5)	13.1 (25.6)
1	1	0.5	1	1.00 (0.00)	1.00 (0.00)	1.00 (0.04)	0.55 (0.50)	0.98 (0.13)	4.4 (5.9)	10.6 (49.8)
1	1	0.5	0.6	0.99 (0.12)	0.96 (0.19)	0.76 (0.43)	0.41 (0.49)	0.76 (0.43)	2.6 (3.7)	11.0 (27.8)
1	3	0.9	1	1.00 (0.04)	1.00 (0.06)	0.98 (0.13)	0.67 (0.47)	0.97 (0.16)	27.3 (27.3)	68.4 (108)
1	3	0.9	0.6	0.90 (0.30)	0.93 (0.26)	0.73 (0.45)	0.48 (0.50)	0.72 (0.45)	23.5 (21.1)	39.8 (90.2)
1	3	0.5	1	1.00 (0.00)	1.00 (0.00)	0.99 (0.08)	0.62 (0.49)	0.99 (0.12)	11.3 (17.4)	12.2 (30.0)
1	3	0.5	0.6	0.99 (0.10)	0.95 (0.22)	0.76 (0.43)	0.38 (0.49)	0.75 (0.43)	4.1 (6.2)	9.47 (20.6)
5	1	0.9	1	0.99 (0.08)	1.00 (0.00)	1.00 (0.04)	0.95 (0.21)	0.98 (0.13)	7.5 (8.1)	105 (143)
5	1	0.9	0.6	0.79 (0.41)	0.99 (0.09)	0.95 (0.22)	0.88 (0.33)	0.95 (0.23)	46.2 (39.8)	182 (255)
5	1	0.5	1	1.00 (0.00)	1.00 (0.00)	1.00 (0.00)	0.96 (0.19)	0.99 (0.09)	0.0 (0.0)	15.4 (26.1)
5	1	0.5	0.6	1.00 (0.04)	1.00 (0.00)	0.98 (0.14)	0.90 (0.31)	0.97 (0.17)	1.4 (2.6)	80.7 (120)
5	3	0.9	1	0.99 (0.10)	1.00 (0.00)	1.00 (0.04)	0.96 (0.19)	0.99 (0.11)	17.5 (14.5)	303 (317)
5	3	0.9	0.6	0.75 (0.43)	0.98 (0.13)	0.91 (0.28)	0.91 (0.29)	0.90 (0.30)	116 (93)	430 (405)
5	3	0.5	1	1.00 (0.00)	1.00 (0.00)	1.00 (0.04)	0.98 (0.15)	0.99 (0.11)	0.0 (0.4)	41.3 (79.9)
5	3	0.5	0.6	1.00 (0.04)	1.00 (0.00)	0.96 (0.19)	0.92 (0.28)	0.95 (0.21)	3.6 (5.5)	217 (325)

## 7. Discussion

### (a) On the choice of *k*

The combinatorial arrangements used are essentially the partially balanced incomplete block designs introduced 80 years ago in the context of plant breeding trials [6]. Our treatment has thus taken *k* as determined *a priori*, preferably below 15. An alternative would be to draw random sets of size *k* for each variable, leaving the choice of *k* undetermined. The argument against too large a value of *k* is that the precision of the resulting estimates is degraded by the correlations induced when there are many correlated variables among those selected for test.

The following is a simplistic formulation outlining the effect of *k* on the estimated standard errors and ignoring other aspects.

Suppose *Z*_1_, …, *Z*_*k*_ have zero mean, unit variance and are weakly correlated with average correlation $\bar{\rho }$. Then the ratio of the variance of *l*^*T*^*Z*, an estimated effect, to that assuming independence is approximately $1-\bar{\rho }+\bar{\rho }{\left(\Sigma l_{i}\right)}^{2}/\Sigma l_{i}^{2}.$

The worst case is where, if the correlations are all of the same sign, the *l*_*i*_ are approximately equal and the ratio becomes $1+(k-1)\bar{\rho }$. Thus, the calculation of standard errors assuming independence is not unduly distorted so long as this ratio does not exceed 2, that is, the average correlation does not exceed about 1/*k*. If correlations of the order 0.05 – 0.1 are likely to be present, *k* of the order 10–20 is a reasonable choice, justifying the choice described here and in the previous paper.

### (b) Arrangement randomization

Strong reassurance of the security of one's conclusions is given if, upon re-randomization of the arrangement of the variable indices in the cube, the outcome is relatively stable. An unstable outcome would most likely indicate that too severe a reduction has been used. The probabilistic properties set forth in §4 guarantee robustness to re-randomization under idealized conditions provided that the decision rules used are not too severe, and empirical experience with microarray data supports this for continuous responses. Some applications with binary outcome require caution. In particular, when *v* is very large, there may be an appreciable number of relatively low-dimensional sets of variables perfectly predicting the outcome. If a set of variables forms such a separating hyperplane, then so does any larger set. The likelihood would, in such a case, be theoretically unbounded and consequently all sets of *k* variables containing a primitive set of separating variables would be retained, leading to too mild a reduction.

The special features of the procedure with binary responses will not be explored in the present paper. Note, however, that agreement of the outcome with the lasso solution should not be expected with binary responses for the reasons outlined by Cox & Battey [3].

### (c) Further remarks

The analysis discussed in the paper is intended to be largely exploratory. The object of the formal probabilistic discussion is to calibrate the procedure to show how it performs under idealized conditions. It is not aimed to justify an explicit probabilistically based assessment, such as a confidence coefficient, attached to a specific analysis. To develop such an assessment, a Bayesian analysis might be considered. For this, meaningful prior probabilities have to be attached to key unknown features, such as the true number of signal variables. A flat or so-called indifference prior would in this case be inappropriate in that it would put overwhelming probability on large values; a Poisson prior distribution of modest mean might be more suitable. Aspects describing the correlation structure of errors also need explicit formulation. At this stage of the work, we have not followed that route.

## Data accessibility

This article has no additional data. The procedure as described in 2 and the previous paper is implemented in the R package 'HCmodelSets', written by H. H. Hoeltgebaum and available on the Comprehensive R Archive Network.

## References

[1] Tibshirani R 1996 Regression shrinkage and selection via the LASSO. J. R. Stat. Soc. B 58, 267–288.

[2] van de Geer S 2016 Estimation and testing under sparsity. Cham, Switzerland: Springer.

[3] Cox DR, Battey HS 2017 Large numbers of explanatory variables, a semi-descriptive analysis. Proc. Natl Acad. Sci. USA 114, 8592–8595. (10.1073/pnas.1703764114) 28739925 PMC5559019

[4] de Bruijn NG 1981 Asymptotic methods in analysis. New York, NY: Dover Publications Corrected reprint of the third edition.

[5] Copson ET 1965 Asymptotic expansions. Cambridge, UK: Cambridge University Press.

[6] Yates F 1936 A new method of arranging variety trials involving a large number of varieties. J. Agric. Sci. 26, 424–455. (10.1017/S0021859600022760)

